# Supplementary material for: A system-on-chip microwave photonic processor solves dynamic RF interference in real time with picosecond latency
Source: Light Sci Appl. 2024 Jan 9;13:14. doi: 10.1038/s41377-023-01362-5 (PMC10776583; doi:10.1038/s41377-023-01362-5)
Supplement: Supplementary file 1 — supplementary information [file 41377_2023_1362_MOESM1_ESM.docx]

**Supplementary Information for**

**A system-on-chip microwave photonic processor solves dynamic RF interference in real time with picosecond latency**

Weipeng Zhang, Joshua C. Lederman, Thomas Ferreira de Lima, Jiawei Zhang, Simon Bilodeau, Leila Hudson, Alexander Tait, Bhavin J. Shastri and Paul R. Prucnal

**Supplementary text contents:**

1. Photonic packaging and complete system setup
2. Latency of digital electronic processor
3. FPGA design for real-time digital peripheral circuitry
4. Evaluation of update rate on BSS performance by simulation
5. Nonlinearity of MRR weighting

**1. Photonic packaging and complete system setup**

Leveraging the fully integrated signal pathway on-chip, we built a compact, portable, comprehensive system setup for our proposed photonic blind source separation (BSS) processor, as depicted in Fig. S1a. Apart from the encapsulated photonic integrated chip (PIC), the system requires only two additional components, including two lasers (PurePhotonics, PPCL500) and a field-programmable gate array (FPGA, RFSoC4x2, Xilinx). Two coaxial cables connect the outputs of the transimpedance amplifiers (TIAs) to the FPGA for statistical analysis. A grey ribbon cable sends digital commands from the FPGA for programming the tuning currents and biasing voltages output by the digital-to-analogue converters (DACs, LTC2662 and LTC2664, Analog Devices) integrated onto the printed circuit board (PCB). Fig. S1b shows the PCBs that mount the PIC, while Fig. S1c provides a close-up view of the PIC, the TIAs, and the fibre array. The PCB is designed with electroless nickel immersion gold (ENIG) process pads connected to the PIC via wire bonding. The tuning currents and biasing voltages are supplied from the pads on the left, while the bottom and top pads are dedicated to RF input and output, respectively. RF inputs are fed through a two-port SMP connector, routed to a bias-tee (MBT-283, Mini-Circuits), and wire-bonded to the chip. Each output photocurrent from an on-chip balanced photodetector (BPD) is wire-bonded to TIAs (HMC7590, Analog Devices) with two duplicated outputs. The FPGA exploits one for statistical analysis, while the other can serve as the system output. The TIA features a 3 dB bandwidth exceeding 39 GHz and a transimpedance gain of 4 kOhms.


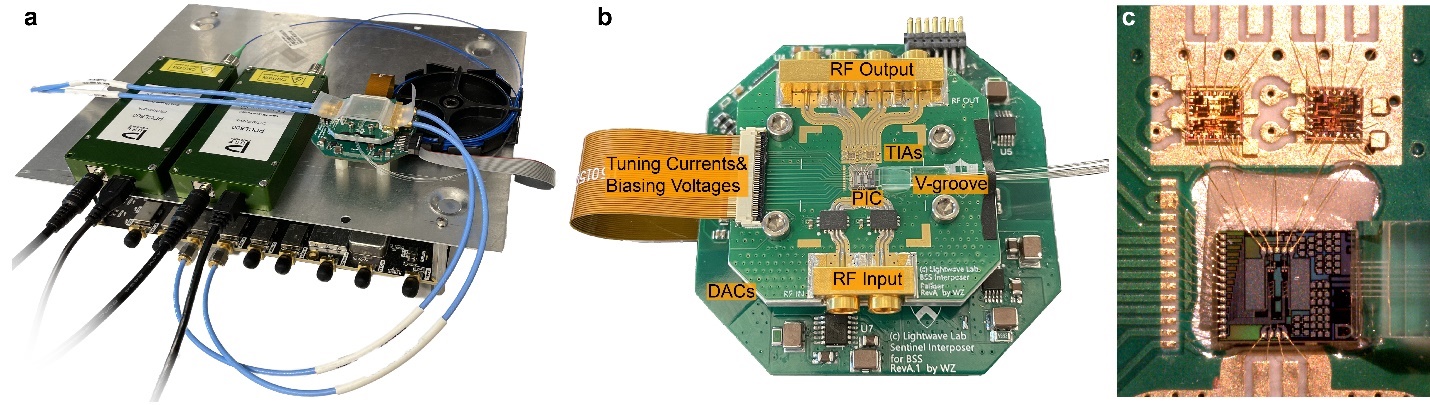


**Fig. S1. Photonic BSS system setup. a** Complete experiment setup. **b** Photo of packaged photonic chip. **c** Close-up view of PIC packaging.

**2. Latency of digital electronic processor**

The core process of BSS is the weighted addition of the received mixtures, which requires a multiplier for each input mixture and a multiple port adder to sum up the products. The multiplier accounts for the majority of the processing latency, as its execution typically requires multiple clock periods and goes up as the bit resolution increases. This mechanism demonstrates the inefficient timing performance of digital electronics that are highly clock-dependent and have an undesirable tradeoff between speed and resolution.

Consider a typical multiplier based on a Wallace tree architecture, which instructs how to multiply two integers using full-adders (FA) and half-adders (HA) to quantify the latency. Comparatively, we may assign the same resolution as the photonic equivalents. Given the typical bit resolution of signal generators and the weighting accuracy of photonic devices, the input mixture and the applied weight have 16-bit and 9-bit representations, respectively. Then, we can determine the six-step, 23-clock-period processing pipeline for the multiplication, as depicted in Fig. S2. Initially, we refer to the two inputs $A$ and $B$, and the $i$th bit in the two numbers as $a_{i}$ and $b_{i}$. Calculate $a_{i}b_{j}$, where $i=1,2,\ldots16$ and $j=1,2,\ldots9$, as illustrated in Fig. S2. This can be easily accomplished with $16\times9=144$ AND gates. Steps 2 through 5 add all these products using FAs and HAs in the order depicted in Fig. S2, with each step requiring one clock period. HAs are used when adding two 1-bit numbers, whereas FAs are used when there are three inputs. The outputs of HA and FA are the sum and the carry. The carry is placed in the adjacent left column for subsequent addition at the more significant bit. In step 6, where the calculation is reduced to two rows, 17 FAs and one HAs are utilised to produce the final result. This step, however, requires 18 clocks because each FA must await the carry result from the adjacent right-column adder. Thus, multiplying a 16-bit integer by a 9-bit integer requires $5+18=23$ clock cycles, which can be executed in parallel for all input mixtures. Finally, it takes one additional clock period to add all multiplication products to complete the weighted addition, for a total of 24 clock periods of latency.


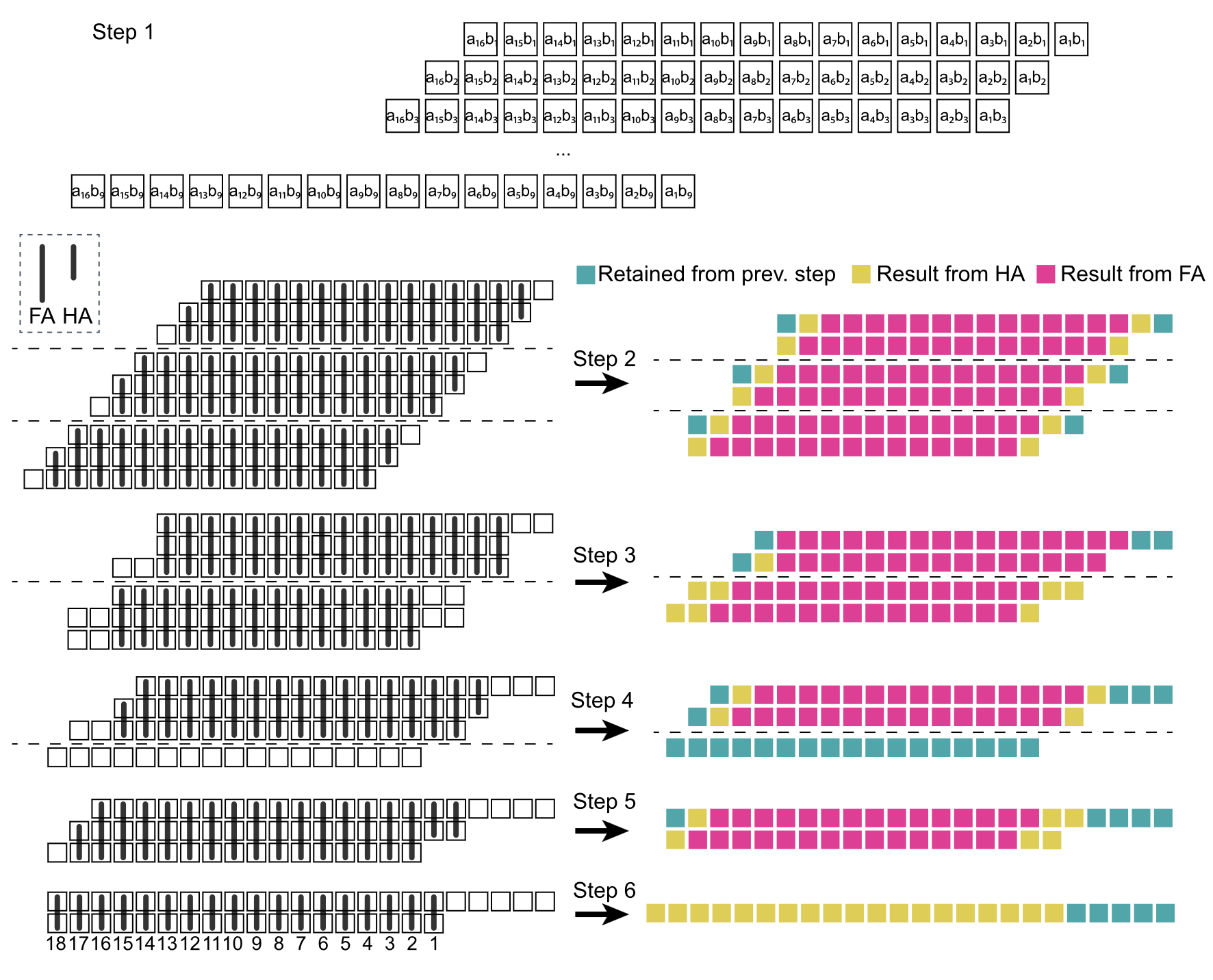


**Fig. S2. Digital electronic multiplier architecture.** This is the multiplication of a 16-bit integer and a 9-bit integer, which consists of 6 steps. Step 1 is bit-wise by AND gates. Steps 2 to 5 utilise full-adders and half-adders, each costing one clock period. Step 6 needs 18 half-adders that operate sequentially and cost 18 clock periods.

Regarding the demonstrated photonic processor, the latency is primarily determined by the signal path length and the speed of light in the medium. The received mixtures enter the path at the MRR modulators depicted in Fig. 2a. The modulated light wave then splits and undergoes processing through two MRR weight banks, ultimately exiting at the balanced photodetector (BPD). The processing latency is the travel time within the photonic chip. Given the total length of the on-chip waveguide is about $1.5 \mathrm{mm}$, the effective index of refraction ($n=2.44$) for the dominant $TE_{00}$ mode, and the speed of light in vacuum ($c_{0}=299792458 m s^{-1}$), the latency can be estimated to be ${1.5}^{-6}/(c_{0}/n)$, or around 12 picoseconds. Notably, this estimated latency is associated with photonic processing alone, which excludes the latency caused by peripheral electrical components and transmission lines, as these elements can exhibit significant variability depending on specific applications. As for the demonstrated setup in this paper, the total processing latency from the "RF In" to "RF Out" connectors (shown in Fig. 2b) is expected to be less than 200 picoseconds.

**3. FPGA design in the real-time digital peripheral circuitry**

In our BSS system, an FPGA is the key enabler for real-time operation and is responsible for many digital peripheral circuitry tasks. The FPGA has four primary functions: analogue-to-digital conversion of the processed output, statistical calculation, high-level optimisation algorithm, and dithering control. The FPGA contains three available tiles, which are a multi-channel RF data converter (RFDC), a block of programmable logic (PL), and an ARM processing system (PS). Using AXI interference, these three tiles can communicate with extremely low latency and high throughput. Fig. S3 depicts the exact architecture. The FPGA chip is a member of Xilinx's RFSoC family, and we use the most recent evaluation board (RFSoC4x2), which is primarily intended for educational use and has a board size of approximately 300 mm (length) by 200 mm (width). Using Vivado (2022.2), the RFDC and programmable logic (PL) FPGA designs are based on the PYNQ (3.7.1) framework. The PS software is developed in C++ for optimal performance.

Regarding the conversion of the analogue output to digital waveforms, the RFDC provides one analogue-to-digital converter with a sampling rate of 4.91 gigasamples per second and a resolution of 14 bits. The ADC generates packets at a frequency of 409.6 MHz and packs 12 samples per packet. Then, these digital packets are processed in the PL block, which utilises all the samples of a given data length $N$ to estimate the second ($\delta^{2}=\Sigma s_{n}^{2}$) and fourth statistical moments ($\mu_{4}= \Sigma s_{n}^{4}$), where $s_{n},n=1,2,\ldots N$ represent each data sample. Then, the PS can easily calculate the kurtosis ($\kappa$) using the formula $\kappa=\mu_{4}/\delta^{4}$. Choosing a sampling length in this design involves a tradeoff between sampling time and noise, as shown in Fig. S3. We choose a sampling size of $2^15$ to optimise performance.


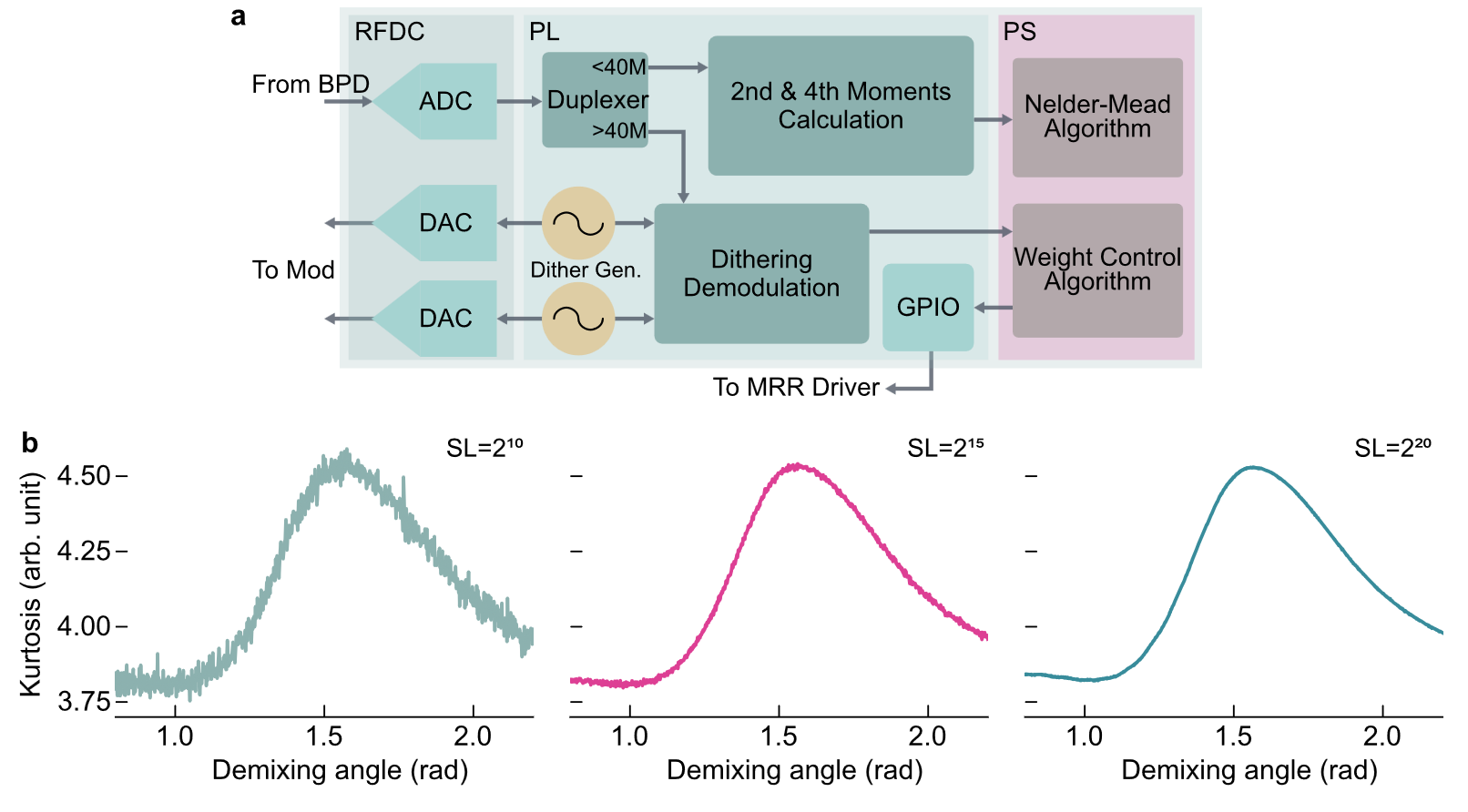


**Fig. S3. FPGA design.** **a** FPGA functional diagram. BPD, balanced photodetector. MZM, Mach-Zehander modulator. GPIO, general purpose input and output. **b** kurtosis measurement at different sampling lengths

This kurtosis measurement is then regarded as the objective function of the optimisation algorithm. The PS runs a program implementing a Nelder-Mead algorithm that iteratively tries new demixing weights to find one associated with the optimal output of the objective function. The PL concludes each iteration by transmitting the demixing weights to the MRR driver via GPIOs. This connection operates at a high baud rate of 50 MHz and utilises the serial peripheral interface (SPI).

In this research, microheaters on top of each ring waveguide are used to thermally tune the micro-ring resonators (MRRs) by applying current. However, inconsistencies between the applied current and the desired weights can result in errors and decrease the bit precision. To address this issue, a dithering method measures precise weights and helps to adjust the current for more accurate weighting. This is achieved using two digital-to-analogue converters in the RFDC to generate dithering signals fed into the signal pathways at the MZMs. The dithering signals are then processed, and lock-in detection is performed at the dithering frequencies, which allows for determining the effective weights applied to each signal channel. Instead of fine-tuning the weights on each iteration, a previously calibrated lookup curve is used to command the applied current based on the desired weights. The optimisation algorithm then uses the measured weights to execute subsequent iterations, and the lookup curve is dynamically updated to account for any deviation between the commanded and measured values. This approach allows for precise weighting without the need for additional iterations.

**4. Evaluation of update rate on BSS performance by simulation**

In an interference environment, the mobility of transceivers can lead to time-variable mixing ratios, as described in Eq. 2, which necessitates a faster execution of each iteration. To address this challenge, we have implemented an FPGA-based digital peripheral with an update rate of 305 Hz. To illustrate its effectiveness, we have emulated a scenario, as shown in Fig. S3a, where a radar altimeter is jammed by a cellular tower.


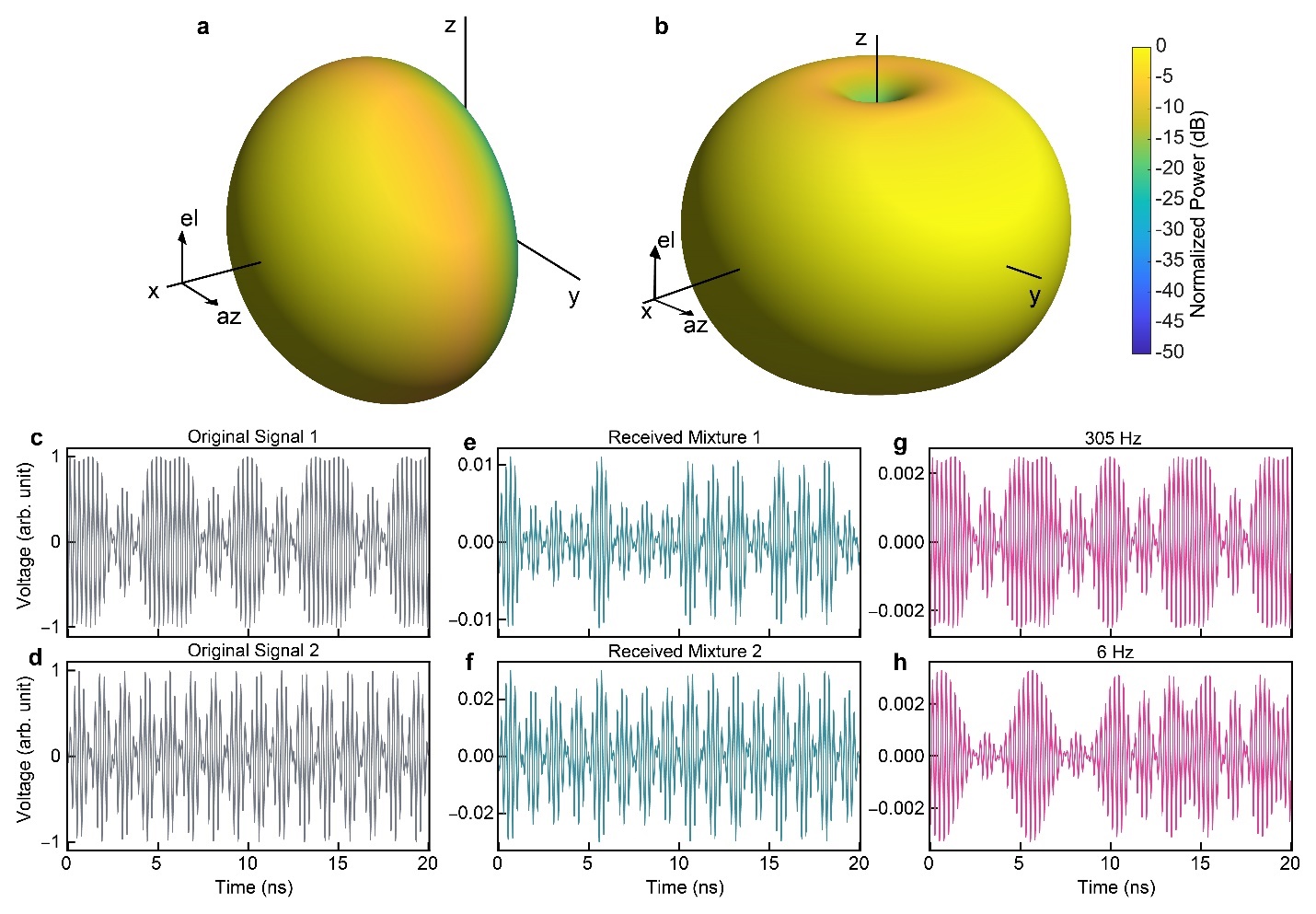


**Fig. S4. BSS Simulation. a** An antenna radiation pattern of Rx and Tx2. **b** Antenna radiation pattern of Tx1. **c** and **d** Original signal waveform. **e** and **f** Received mixtures at $t=10 s$. **g** Recovered signal by BSS with a refresh rate of 305 Hz. **h** Recovered signal by BSS with a refresh rate of 6 Hz.

In this emulation, the radar receiver is a 2x2 MIMO antenna composed of two identical sub-antennas, which are directional and have a cosine radiation pattern, as shown in Fig. S4. The sub-antennas are distinguishable by their orthogonal polarisation angles: $e_{rx1}=(\sqrt{2}/2,-\sqrt{2}/2,0)$ and $e_{rx2}=(-\sqrt{2}/2,\sqrt{2}/2,0)$. As per the radar altimetry, another antenna (Tx2) is onboard to transmit the radar signal in the format of FMCW towards the ground. This transmitter has the same radiation pattern as the receiver and a polarisation angle in the exact middle of the two slants of the receiver, which is $e_{tx2}=(1,0,0)$. Another antenna transmits interference signals from a fixed location on the ground (Tx1). This antenna is omnidirectional, vertically polarised, and has an ideal dipole-typed radiation pattern, as shown in Fig. S4. The receiver and the onboard transmitter stay relatively stationary. The radar signal transmitted from Tx2 completes a round-trip between the aeroplane and the ground, which is constant in power and phase. However, as shown in Fig. 4a and b, the aeroplane movement can vary the distance between the Rx and Tx1 and the related antenna gains. The change in the incident angle, the polarisation angle, and the non-isotropicity of the radiation patterns contribute to the gain variation. In the real world, this gain variation is difficult to predict and requires the blindness feature offered by BSS.

To carry out the emulation, two transmitted signals are generated, as shown in Fig. 4c and d. These signals are BPSK-modulated with the same carrier frequency of 4 GHz and data rate of 800 MHz but have different bit patterns. The impact of the update rate on recovery performance is evaluated by testing the BSS algorithm at three different update rates. As shown in Fig. S4g and h, only a BSS processor with real-time operability, which has a refresh rate of 305 Hz, can effectively track and compensate for the variance of the mixing ratios and achieve consistent signal recovery during the tested period. Slow BSS processors with update rates of 6 Hz and below can result in significant errors and decreased signal-to-interference ratios, highlighting the importance of real-time operability in addressing dynamic interference.

**5. Nonlinearity of MRR weighting**

The demonstrated processing link may introduce signal degradation stemming from the nonlinearity inherent to the EO modulation and MRR weighting. Additionally, noise introduced by the photodetector and the transimpedance amplifier can further exacerbate the degradation.

To examine this, we conducted an ancillary experiment to measure the Spurious-Free Dynamic Range (SFDR) in both pre and post-photonic processor intervention. In this experiment, the wireless transmission path was bypassed, and instead, a single-tone signal (4 GHz) output from a signal generator (M8196A, Keysight) was channelled directly to one of the on-chip modulators. Subsequently, all the weight weights were set to 1, thereby ensuring the output signals have the maximal amplitudes. Then, we recorded the processed output from the on-chip photodetector by a scope (DPO73004SX, Tektronix) and calculated the signal spectrum as shown in Fig. S5 that indicating SFDRs of 25.5 dBc and 11.5 dBc before and after the processing, respectively. It is worth noting that the additional spectral noise components, aside from the signal harmonics, are primarily attributed to the mismatch in the sampling rates of the signal generator, oscilloscope, and the signal frequency—rated at 88 GSPS, 100 GSPS, and 4 GHz respectively, through the associated aliasing effects. Fortunately, these harmonics remain significantly lower in power than the signal of interest and its major harmonics.


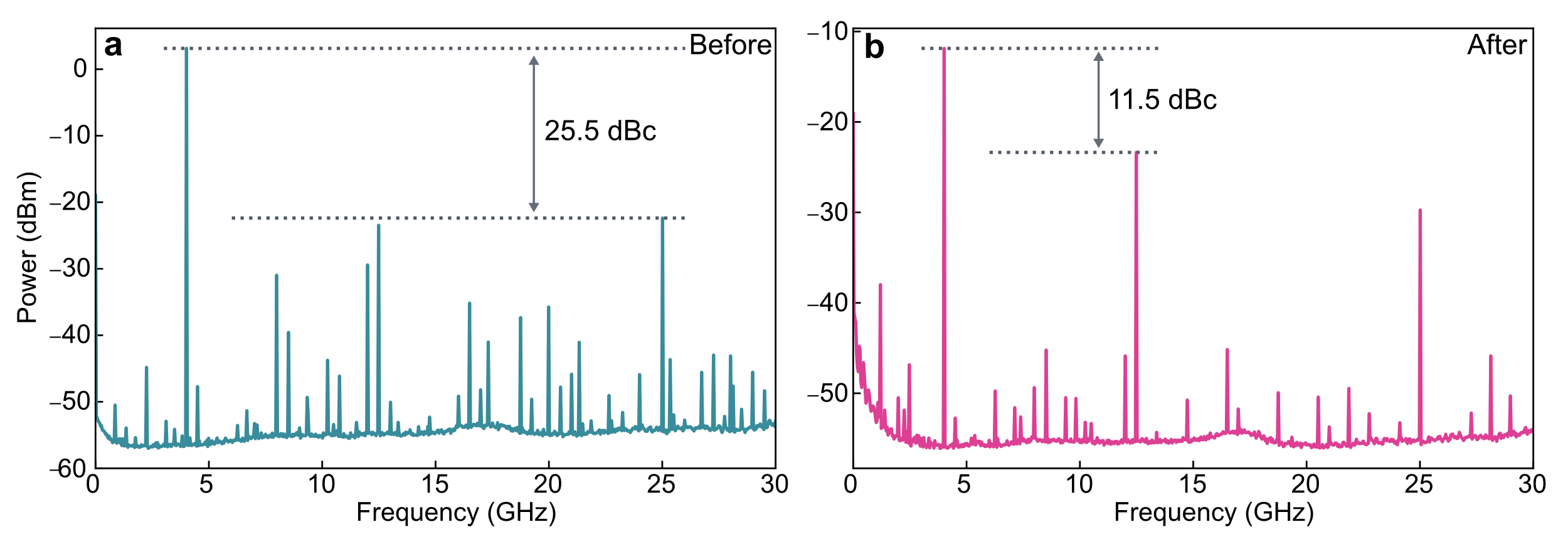


**Fig.** **S5 Signal fidelity degradation by MRR nonlinearity.** Spurious-Free Dynamic Range before (**a**) and after (**b**) processed by the proposed photonic processor. Original signal is a 4 GHz single-toned sinusoid wave.

The nonlinearity of an MRR reflects in both the non-flat amplitude response and phase jump at the centre of the resonance, and in our setup, the latter one actually introduces minimal distortion. This is because the balanced detection scheme used by the MRR weight bank only requires MRRs to be predominantly tuned to the range within one side of the resonance, where the phase variations are markedly less pronounced.

Nevertheless, the deviation from a flat amplitude response does introduce nonlinearity, and it is intrinsically related to the Q-factor and the signal bandwidth. A steeper and narrower transmission profile emerges with a heightened Q-factor. Consequently, different spectral components within the widened spectrum of a modulated laser light can undergo disparate filtering extents, amplifying distortion. To mitigate excessive distortion, our MRRs were carefully designed to maintain an optimised Q-factor of 6000. This design was proven to work in our previous work; utilising similarly designed MRRs, we successfully cancelled interference up to 20 GHz.

Each MRR spans approximately 37 GHz, with a gap of 200 GHz between successive MRRs. Maintaining the current resonance frequency, the MRR transmission width can potentially be expanded up to 5 times (300/37) without spectral interference. With the free spectral range (FSR) of each MRR being around 1 THz (8.5 nm), and by evenly distributing the MRRs' resonating frequencies within an FSR, the MRR spectral width can potentially be augmented about 27 times (1000/37) its current value. This enhancement could be even more pronounced if the setup requires fewer MRRs, as is the case with our two-MRR configuration.


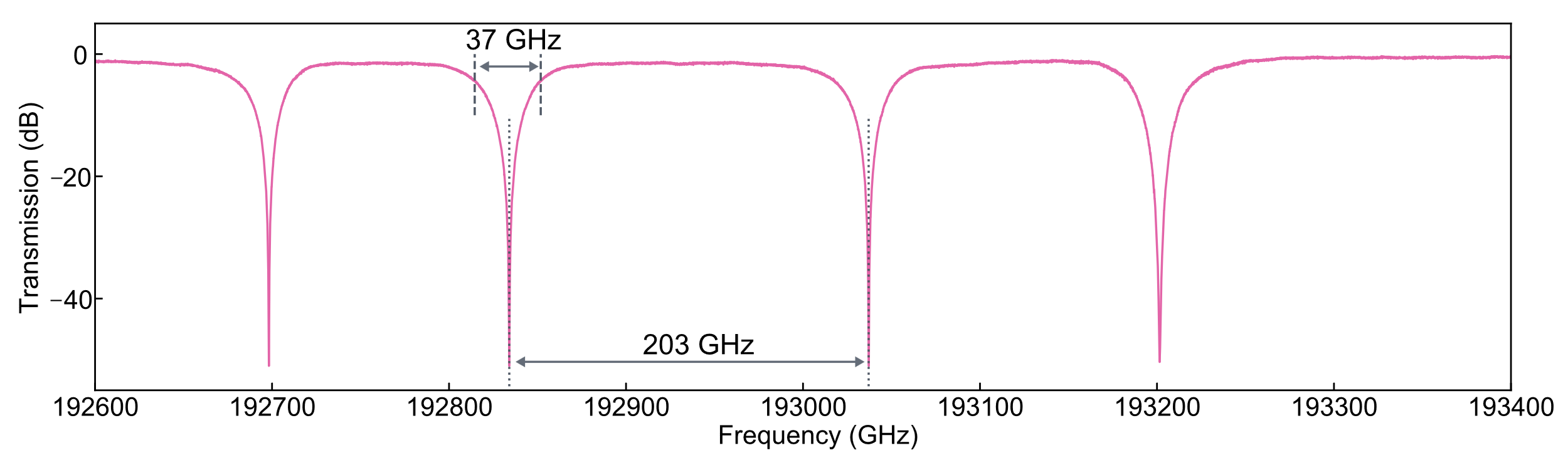


**Fig. S6 MRR spectrum.** Each MRR has a full width at half-maximal (FWHM) of 37 GHz and a free spectral range of 1 THz. The spectral spacing between adjacent MRRs is about 203 GHz.

We are also exploring an alternative solution that hinges on reconfiguring the signal processing pathway, as visualised in Fig. S7. The intrinsic cause of the distortion issue lies in the broadened laser linewidth after modulation, which is then subjected to non-linear filtering. If the unmodulated light is pre-filtered using the MRR and subsequently modulated, we can sidestep the non-linear filtering effects. This approach is currently under preliminary evaluation and is slated for experimental testing in our subsequent works.


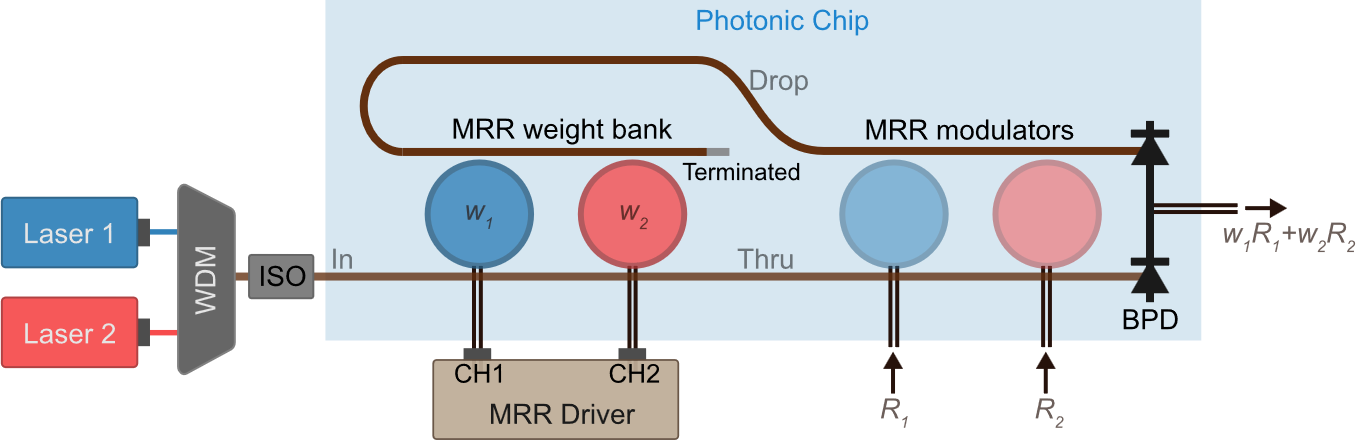


**Fig. S7 A new layout for broadband performance.** Unmodulated laser lights are first weighted by an MRR weight bank, which allows control of the power distribution from the In port to the thru and drop port. Subsequently, an array of PN-junction-typed MRR modulators, positioned between the waveguides corresponding to the thru and drop ports, modulate input broadband RF signals onto these pre-weighted but still unmodulated lights. Noting that a portion of the light can reflect and propagate in the reverse direction along the waveguides. To address this, isolators and absorbers are incorporated, as depicted in the provided layout. Then, an array of MRR modulators (PN-junction typed) sitting in between the waveguides corresponding to the thru and drop ports can modulate input broadband RF signals onto the weighted but unmodulated lights. Noting that the partial lights can be reflected and reversely propagated along the waveguides. Therefore, isolators and absorbers need to be used, as shown in the layout. ISO, isolator. BPD, balanced photodetector.
